# Comparative analysis of cell culture and prediction algorithms for phenotyping of genetically diverse HIV-1 strains from Cameroon

**DOI:** 10.1186/1742-6405-6-27

**Published:** 2009-11-25

**Authors:** Viswanath Ragupathy, Jiangqin Zhao, Xue Wang, Owen Wood, Sherwin Lee, Sherri Burda, Phillipe Nyambi, Indira Hewlett

**Affiliations:** 1 Laboratory of Molecular Virology, Center for Biologics Evaluation and Research, Food and Drug Administration, Bethesda, MD 20892, USA; 2 Department of Pathology, NYU School of Medicine, 550 First Avenue, Medical Sciences Building, 5th Floor, New York, NY 10016 423, USA

## Abstract

**Background:**

With the advent of entry inhibitors, monitoring of viral tropism in the clinical setting is important. Conventional methods are cell-based and lengthy, therefore V3 sequence based prediction algorithms are becoming increasingly attractive as monitoring tools. Here we report a comparative analysis of viral tropism of strains circulating in Cameroon where diverse and emerging variant strains are prevalent.

**Methods:**

Viruses were isolated from 17 HIV positive individuals from three cities in Cameroon. Ghost cell lines expressing either CCR5 or CXCR4 with CD4 or CD4 alone (NIH AIDS Reagent Program) were used to determine co-receptor usage. HIV replication was determined by measuring p24 antigen levels. Plasma viral load (VL) was determined using the Versant bDNA assay. Nucleotide sequencing was performed on the V3 region and sequences were edited, aligned and translated into amino acids as described in the algorithm. Bio-informatics tools based on the 11/25 and charge rule were used to predict co-receptor usage.

**Results:**

The majority of patient isolates in our study were CRF02_AG or CRF02_AG containing recombinants. Tropism of these complex viruses based on the cell culture assay was determined to be R5 in 15/17 (88.2%) patients. However, two patient isolates were dual tropic R5X4 and had drug-specific mutations. Of these two patients, one was on antiretroviral treatment with a VL of 20,899 copies/ml and the other was drug-naïve with 141,198 copies/ml. Genotype based prediction was overall in good agreement with phenotype for R5 viruses, where 93% (14/15) of results were comparable, dual tropic viruses being reported as X4 viruses by prediction.

**Conclusion:**

Our results indicate that most HIV strains in Cameroon were R5 tropic and some harbored drug-resistant mutations. V3 sequence based prediction compared well with cell based assays for R5 strains and may be useful even in settings where highly diverse strains are prevalent.

## Findings

Human Immunodeficiency virus type 1 (HIV-1) enters the cell by a multistep process that involves CD4 binding and the use of co-receptors CCR5 or CXCR4. Co-receptor usage in many cases correlates with disease pathogenesis and progression [1,2]. Furthermore, changes in viral tropism occurs in many HIV positive individuals over time, indicated by a shift in co-receptor use from CCR5 to CXCR4 which has been shown to generally correlate with increased disease progression [3]. Some viruses are capable of using both co-receptors and are termed dual tropic or R5X4 viruses. In the era of antiretroviral therapeutics, co-receptor antagonists are now in use for treatment of HIV infected individuals [4], and it therefore becomes necessary to identify strains circulating in a given population or region on the basis of their tropism. This should be helpful to clinicians by providing additional information for better management of disease.

Currently there are two methods in practice for co-receptor determination a) bio informatics tools based on V3 sequence to predict co-receptor use and b) transfected cell culture based methods. The latter method is widely used in many clinical settings but is labor intensive and time consuming. Prediction of co-receptor usage based on V3 sequence data on plasma viral RNA may be a useful alternative tool to assist clinicians in situations where virus culture based phenotyping methods that rely on isolation of peripheral blood mononuclear cells (PBMC) from patient specimens may not be practical while also being labor-intensive and time consuming.

The present investigation was aimed at characterizing genetically diverse HIV-1 strains circulating in Cameroon in terms of co-receptor usage and comparing cell culture based methods with V3 sequence based prediction algorithms for virus phenotyping and co-receptor usage of complex, emerging HIV strains.

Virus isolates (n = 17) were obtained from patients attending clinics in three cities in Cameroon - Bamenda, Limbe and Buea. Demographic information was collected in the Performa and analyzed. Viruses were propagated in PBMC derived from buffy coats and cell free viruses stored in liquid nitrogen for subsequent analysis. Ghost cell lines (Human osteosarcoma cells) expressing CCR5, CXCR4 with CD4 or CD4 alone (received from NIH AIDS Reagent Program) were used to determine co-receptor use. Briefly, cells were seeded at a concentration of 10e5 cells/well in a 24 well plate. After 24 hours, cells were infected with 5 ng of p24 antigen of different HIV strains, incubated at 37°C with 5% CO2 for 2 hours, washed thoroughly and cultured in MEM media with10% FBS and antibiotics. Appropriate controls included uninfected cells, and cells treated with co-receptor antagonists TAK 779 (9.14 μmol/ml) and AMD 3100 (100 ng/ml) to block CCR5 and CXCR4. Culture supernatants were harvested at days 4 and 8 and HIV replication was determined by measuring p24 antigen levels using the Perkin Elmer kit (Cat No: NEK050B).

Viral RNA was isolated using the QIAGEN (Cat No: 52906) viral RNA extraction kit The V3 region was amplified by nested PCR and sequenced using primers *ED31 5'-CCTCAGCCATTACACAGGCCTGTCCAAAG *and ES8 5'-CACTTCTCCAATTGTCCCTCA and sequences were edited, aligned in Clustal program and translated into amino acids as described in the algorithm. Plasma VL was determined using the Versant HIV RNA 3.0 Assay (bDNA; Siemens, IL) for 15 of the 17 samples studied.

In our analysis, we used 11/25 and charge rule bio-informatics tool to predict co-receptor usage [5]. The positively charged amino acids at 11/25 and net charge >5 predict CXCR4 in the V3 loop aligned against a consensus sequence although other positions may also be important.

The 17 viruses studied were obtained from nine males and eight females whose ages ranged from 37-54 Yrs for men and 20-60 Yrs for women. Because 9/17 (53%) of the patients were on anti-retroviral therapy with Triamune (3TC/d4T/NVP) or a combination of lamivudine(3TC)/Stavudine(d4T) or lamivudine(3TC)/Nevirapine(NVP), we examined the pol sequences of their viral isolates for evidence of drug specific resistance mutations. Genotyping revealed that 5/9 (55.5%) had drug specific mutations. Of these 5 patients, 2 had drug resistance for all classes of RT antiretroviral drugs NRTI (A62V, K65R, T69I, V75I, F77L, F116Y, Q151M, M184V/I) and NNRTI (V90I, V108I, Y181C, Y188L, M184I) and 3 had one or more drug specific mutations, K103N, Y181C and G190A in the RT region. HIV disease progression is generally associated with high VL and X4 phenotype, therefore we determined plasma VL for both treatment-experienced and drug naïve patients. Those who received ARV therapy had a VL range of <75-28987 copies/ml while drug naive patients ranged from <75-141,198 copies/ml.

Since multiple, diverse strains are responsible for the HIV epidemic in Cameroon, we analyzed viral sequences of the strains to identify genetic subtype. Phylogenetic analysis of partial sequences of gp41, p17 and pol region revealed that 53% belonged to CRF02_AG, 6% subtype F2 and 41% were Unique Recombinant Forms (URFs) (Table 1). It is interesting to note that majority of viruses were recombinants of CRF02_AG with gene segments of other HIV CRFs and subtypes suggesting that newly emerging HIV strains in Cameroon may be recombinants of the predominant CRF strain with other lesser CRF variants contributing to the evolving diversity of HIV in this region. The tropism of these complex viruses based on cell culture experiments was determined to be R5 for 15/17 (88.2%) strains (Table 1). A possible explanation for the predominance of R5 tropism observed with the CRF02_AG viruses we studied may be attributed to the subtype A of the env gene segment in these strains and it has been reported earlier [6] that subtype A strains generally show CCR5 tropism. In our study virus isolates from two patients (06CMLPH02MG; 60CMBDSH05) were found to be dual tropic R5X4 and their genotypes were assigned as URFs. Thus, most representative viruses circulating in the regions we studied were classified as R5 viruses. As entry inhibitors for CCR5 and CXCR4 co-receptors are currently being used for HIV treatment, fast and reliable methods for determination of viral tropism will be of value to clinicians. Many previous studies have shown that V3 sequence based prediction algorithms can be comparatively rapid and reliable for population studies [7-10]. However, the ability of such tools to accurately predict co-receptor usage of viruses in a population that harbors genetically diverse HIV strains needs evaluation in order to determine their appropriateness and suitability for determination of viral tropism.

**Table 1:** Comparison of genotypic prediction vs phenotype

Sample ID	Genotype	Prediction	Ghost cells Assay
06CMARC007	URF	CCR5	CCR5
06CMARC009	CRF02	--	--
06CMARC036	CRF02	--	--
06CMARC058	CRF02	--	--
06CMLPH01OJ	URF	--	--
06CMLPH03VJ	CRF02	--	--
06CMLPH11TT	URF	--	--
06CMLPH016SL	CRF02	--	--
06CMLPH17HT	CRF02	--	--
06CMLPH19CM	URF	--	--
06CMLPH20SL	CRF02	--	--
06CMBDHS019	F2	--	--
06CMBDHS024	URF	--	--
06CMBDHS064	CRF02	--	--

07CMLPH128	CRF02	CXCR4	CCR5
06CMLPH02MG	URF	CXCR4	CXCR4/CCR5
06CMBDSH05	URF	CXCR4	CXCR4/CCR5

Columns indicate sample ID's and their corresponding HIV genotype from partial sequences (gp41/p17/pol), its tropism by prediction and cell culture assay.

In our study, V3 sequence based prediction was found to be in good agreement with phenotype, where 93% (14/15) of results were comparable (Table 1). One patient isolate (07CMLPH128) was an R5 virus but genotype prediction scored it as X4 tropic because of mutations in the V3 region and a net charge of 8. For these field isolates the combined 11/25 and charge rule based prediction was found to be most appropriate when compared with other methods (data not shown). Similar observations were reported in a study with the combined use of the 11/25 and net charge rules [5]. Furthermore, eight web based prediction algorithms when used individually to determine viral tropism, had a low sensitivity and specificity for non-B subtypes. However, for clade B viruses, only PSSM_X4R5 _and geno2pheno yielded good sensitivity and specificity when compared with other algorithms [11]. Our study findings suggest that direct V3 sequencing may provide an alternative to phenotypic assays for assessing HIV-1 tropism. As reported earlier [12] dual tropic viruses could be under estimated and we observed similar findings in our data set. Our findings are in good agreement with earlier observations for R5 viruses that prediction methods based on V3 sequencing are comparable with the phenotypic method suggesting their potential applicability in clinical settings for the future.

Another observation in our limited study is that among those who received antiretroviral therapy in our study population, two individuals (ID = 06CMBDSH05; 06CMARC007) had resistance to all classes of RT inhibitors with the first strain showing R5X4 and the second, R5 tropism. Viruses from both individuals were classified as URFs having CRF02_AG with CRF11 for 06CMBDSH05 and a small fragment of subtype B in the gag region for 06CMARC007 (full length, unpublished data). A third individual (ID 06CMLPH02MG) had never received antiviral therapy but his viral phenotype was R5X4 with VL being 141,198 copies/ml suggesting potential advancement of disease accompanied by X4 tropism and drug resistance. Interestingly the virus was a URF composed of the predominant strain in Cameroon, CRF02_AG with a minor CRF37, suggesting that complex recombinants emerging in this region could likely be dual tropic viruses. Larger studies and data sets are needed from these regions to further substantiate these findings and to understand the complexity of the emerging diversity of HIV in this region of high diversity.

Finally, although higher frequencies of drug resistance in patients harboring X4 viruses have been reported recently [13], in our study of the 5 individuals on therapy, we identified drug resistant mutations in four with R5 tropic and one with X4 tropic viruses. Studies with larger sample sizes are required to determine the impact of drug resistance on tropism and whether emergence of new recombinants, drug resistance and viral tropism play a major role in the spread and diversification of HIV strains in Cameroon. In conclusion, our results, based on a limited number of specimens and viruses isolated from PBMC sampled for phenotypic and genotypic studies indicate that even in a geographic region where highly complex viruses circulate, the V3 prediction algorithm compared favorably with cell culture for R5 viruses that were predominant in this region. These findings suggest that V3 sequence based co-receptor prediction may potentially be an alternate tool to cell culture assays for phenotypic characterization of emerging, new and diverse HIV strains in a population where genetic diversity is high and continuing to evolve.

## Competing interests

The authors declare that they have no competing interests.

## Authors' contributions

VR have made substantial contributions to conception, design, analysis and interpretation of data, JZ helped with sequence data analysis, XW with tissue culture work, OW & SL assisted with virus culture from stocks, SB & PN provided the viruses for the work, PN and IH have been involved in drafting the manuscript or revising it critically for publication, IH provided supervision for the project.
